# Immunohistochemical and western blot expression of MMPs, TIMPs, and cytokeratin 10 in feline squamous cell carcinoma

**DOI:** 10.3389/fvets.2026.1787600

**Published:** 2026-04-10

**Authors:** Paul Tutu, Gennaro Altamura, Florentina Daraban Bocaneti, Ozana Maria Hritcu, Aurelian-Sorin Pasca, Mihaela Anca Dascalu, Loredana Horodincu, Oana Irina Tanase, Mihai Mares, Giuseppe Borzacchiello

**Affiliations:** 1 “Ion Ionescu de la Brad” Iasi University of Life Sciences, Iasi, Romania; 2 Department of Veterinary Medicine and Animal Production, University of Naples Federico II, Napoli, Italy

**Keywords:** squamous cell carcinoma, cytokeratin, feline, matrix metalloproteinases, papillomavirus

## Abstract

**Background:**

Feline oral and cutaneous squamous cell carcinoma (SCC) are aggressive neoplasms characterized by high local invasiveness. Given its spontaneous occurrence and molecular similarities, feline SCC represents a robust comparative model for human head and neck squamous cell carcinoma (HHNSCC). This pilot study aimed to characterize the expression of metalloproteinases (MMPs), their tissue inhibitors (TIMPs), and cytokeratin 10 (CK10) in relation to Papillomavirus (PV) status, and to explore the viral-molecular relationship in feline oncology.

**Methods:**

Ten feline SCC samples (3 oral and 7 cutaneous) were analyzed. PV DNA was detected using PCR with degenerated primers FAP59/FAP64. Protein expression of MMP-2, -9, -13, -14, TIMP-2, -3, and CK10 was analyzed and quantified via immunohistochemistry (IHC) (*H*-score) and validated through Western blot analysis. Statistical correlations were determined using the Spearman rank correlation coefficient, and statistical differences between groups were assessed via Mann–Whitney *U* test.

**Results:**

PV DNA was identified in 6/10 samples. IHC analysis revealed that MMPs (-2, -9, -13, -14) exhibited stronger cytoplasmic and nuclear immunostaining compared to the more restricted signals of TIMPs and CK10, showing a strong inverse correlation with MMP-2, linking invasiveness to dedifferentiation. Significant positive correlations were found between MMP-9/TIMP-3 and MMP-2/MMP-9. TIMP-2 expression was significantly higher in PV-negative samples. Western blot confirmed these results, showing consistent bands.

**Conclusion:**

This pilot study provides the first characterization of MMPs, TIMPs, and CK10 in feline SCC, establishing a foundation for future research into feline PV species and reinforce the value of the feline model in comparative oncology.

## 1 Introduction

Feline cutaneous and oral squamous cell carcinoma (CSCC, OSCC) are common and aggressive tumors in cats, often linked to PV infection (1–3). SCC accounts for about 15% of cutaneous and 60%–75% of oral tumors in felines (4, 5). Beyond its veterinary importance, feline OSCC represents a robust model for HHNSCC due to similarities in clinical behavior, molecular profile, and risk factors (6, 7). This comparative pathology approach suggests that investigating molecular mechanisms in the feline model may directly benefit oncological progress in both veterinary and human medicine (8).

MMPs, particularly MMP-2 and MMP-9, and their inhibitors regulate tumor invasion and progression, with imbalances linked to cancer aggressiveness (9, 10). Similar mechanisms have been observed in bovine fibropapillomas associated with papillomavirus infection (11, 12).

CK10 marks epithelial differentiation, while its loss is correlated with tumor progression and aggressiveness in SCC (13, 14). An inverse relationship between MMP-9 and CK10 expression in tumorigenic keratinocytes suggests that decreased differentiation is linked to higher invasive potential (15). Although MMPs are known markers of invasion (16), this specific panel (MMP-2, 9, 13, 14, TIMP-2, 3, and CK10) has never been investigated in relation to PV status in cats.

Therefore, this pilot study aimed at evaluating the immunohistochemical and Western Blot expression of MMP-2, -9, -13, -14, TIMP-2, -3, and CK10 in a cohort of feline SCCs (cutaneous and oral), correlating these molecular profiles with PV status (detected by PCR). Our objective was to identify co-expression patterns that could serve as indicators of virus-influenced tumor progression.

## 2 Methods

### 2.1 Samples

A total of 10 SCC samples collected from cats were analyzed. Five cases were retrieved from the Infectious Diseases Clinic, Faculty of Veterinary Medicine, Iasi, Romania, where each sample was divided into two parts: one part stored at −80 °C for biochemical assays and the other fixed in 10% buffered formalin for histopathological analysis. The remaining five cases were retrieved from the archives of the Anatomical Pathology Department of the same institution, and included only as paraffin-embedded samples. For molecular biology studies, 20 μm-thick sections were cut from each paraffin block using separate microtome blades and collected in sterile tubes to prevent cross-contamination.

### 2.2 PCR

Genomic DNA was isolated from the SCC samples using the Qiagen DNA Mini Kit following the manufacturer’s instructions. PV DNA detection was performed by conventional PCR targeting a conserved ~478 bp fragment of the L1 gene. The analysis utilized the degenerate primers FAP59 (5′-TAACWGTIGGICAYCCWTATT-3′) and FAP64 (5′-CCWATATCWVHCATITCICCATC-3′), as described by (17).

The PCR amplification was carried out in a final volume of 20 μL containing: 5 μL of extracted DNA, 1X PCR buffer (Invitrogen, Platinum II Hot-Start Green PCR Master Mix) and 0.75 μM of each primer. The thermal cycling conditions consisted of an initial denaturation at 94 °C for 10 min, followed by 45 cycles of denaturation at 94 °C for 90 s, annealing at 50 °C for 90 s, and extension at 72 °C for 90 s, with a final extension step at 72 °C for 5 min.

To ensure the reliability of the results, a negative control (nuclease-free water) was included in each run. The amplified products were visualized by electrophoresis on a 2% agarose gel stained with SYBR Safe DNA Gel Stain (Invitrogen) and compared against a 100 bp DNA ladder (Invitrogen). Digital representation of the gel was captured and analyzed using the iBright FL1500 Imaging System (Invitrogen).

### 2.3 Histopathology

The samples were fixed in 10% buffered formalin and then embedded in paraffin for routine histological processing and staining. Light microscopy evaluation was performed according to the guideline proposed by (18) using hematoxylin and eosin staining.

### 2.4 Immunohistochemistry

Paraffin-embedded sections from 10 SCC samples were subjected to immunohistochemistry using the streptavidin-biotin-peroxidase method (Novolink Polymer Detection System; Leica Biosystems, Newcastle, United Kingdom), following the protocol described by (11). Primary antibodies against MMP-2 (PA5-16504, Thermo Fisher), MMP-9 (PA5-27191, Thermo Fisher), MMP-13 (MA5-14238, Thermo Fisher), and MMP-14 (PA5-104459, Thermo Fisher) were applied for 1 h at room temperature. Anti-TIMP-2 (MA5-12207, Thermo Fisher) and anti-TIMP-3 (PA5-70423, Thermo Fisher) antibodies were incubated overnight at 4 °C, as described by (11, 12, 19). Anti-CK10 (MA5-42858, Thermo Fisher) was applied overnight at 4 °C at 1:200 dilution. Diaminobenzidine was used to visualize specific immunoreactivity. The protocol included bovine cutaneous fibropapilloma samples as positive controls, which have been shown to express these MMPs and TIMPs (11, 12), while for the negative controls the primary antibodies were omitted and substituted with phosphate-buffered saline. Immunoreactivity was analyzed using QuPath 0.5.1 open-source software, with digital image analysis performed on 3 different images at 40× magnification. The *H*-score was calculated using the method described by (20): (1 × percentage of weak staining) + (2 × percentage of moderate staining) + (3 × percentage of strong staining) within the target region, yielding scores from 0 to 300.

### 2.5 Western blotting

Biochemical analysis was conducted on 5 tissue samples of feline SCC. Protein extraction, electrophoresis, blotting, and background blocking were carried out following the protocol described by (21). The membranes were incubated with the following primary antibodies: anti-MMP-2 (1:500), anti-MMP-9 (1:1000), anti-MMP-13 (1:1000), anti-MMP-14 (1:1000), anti-TIMP-2 (1:500), anti-TIMP-3 (1:500), and anti-Cytokeratin10 (1:500). After washing with TBS-0.1% Tween buffer, HRP-conjugated secondary antibodies, horse anti-mouse (Cell Signaling Technology #7076S) and goat anti-rabbit (Cell Signaling Technology #7074S), were applied for 1 h at room temperature. Protein bands were detected via enhanced chemiluminescence (ECL, Bio-Rad) using a ChemiDoc gel scanner (Bio-Rad). To ensure comparable protein loading and for normalization of densitometric data, β- actin was used as the internal reference protein (housekeeping protein), membranes were stripped and incubated with mouse anti-β-actin antibody (C-2: sc-8432, Santa Cruz) at a 1:500 dilution. Densitometric quantification of proteins was performed using Image Lab software (Bio-Rad), with protein levels normalized to β-actin and expressed as densitometric ratios.

### 2.6 Statistical analysis

Statistical analysis was performed using SPSS version 26.0. Given the small sample size (*n* = 10) and the exploratory nature of this pilot study, non-parametric tests were employed as they do not assume a normal distribution of the data.

To evaluate differences in protein expression (*H*-scores) between groups (PV+ vs. PV− and cutaneous vs. oral samples), the Mann–Whitney *U* test was applied. Potential biological relationships and co-expression patterns between the investigated markers were assessed using the Spearman’s rank correlation coefficient, and the corresponding correlation matrix was visualized as a heatmap generated with GraphPad Prism v10.6.1.892.

Descriptive statistics are presented as mean ± standard deviation (SD). For all analyses, a *p*-value of *p* < 0.05 was considered statistically significant. Due to the limited cohort size, results where *p* > 0.05 but showing visible differences in distribution were interpreted as trends rather than definitive conclusions.

## 3 Results

### 3.1 Samples

Nine out of the 10 samples collected were from females and 1 from a male. The majority of patients (*n* = 8) were of the European breed, with two cases belonging to the Persian breed. All skin samples collected were from ear formations (*n* = 7), while oral samples were from portions of the oral mucosa (*n* = 2), and tongue (*n* = 1).

Regarding the age, the cats were classified according to well-known life stages: kitten (up to 1 year), young adult (1–6 years), mature adult (7–10), senior (more than 10 years) (22). The predominant cats age diagnosed with neoplastic diseases was senior (*n* = 4), followed by young adult (*n* = 5) and mature adult (*n* = 1) (Table 1).

**Table 1:** Clinical data and laboratory results obtained on the tested samples.

Sample	Age	Sex	Breed	Tumor location	Histopathology	PCR
CSCC1	1 year	F	European	Ear skin	CSCC	−
CSCC2	3 years	F	European	Ear skin	CSCC	+
CSCC3	5 years	F	European	Ear skin	CSCC	+
CSCC4	11 years	F	European	Ear skin	CSCC	−
CSCC5	11 years	F	Persian	Ear skin	CSCC	−
CSCC6	14 years	F	European	Ear skin	CSCC	+
CSCC7	5 years	F	European	Ear skin	CSCC	+
FOSCC1	8 years	F	European	Oral mucosa	OSCC	−
FOSCC2	12 years	F	Persian	Tongue	OSCC	+
FOSCC3	2 years	M	European	Oral mucosa	OSCC	+

### 3.2 Histopathology

**Figure 1:**
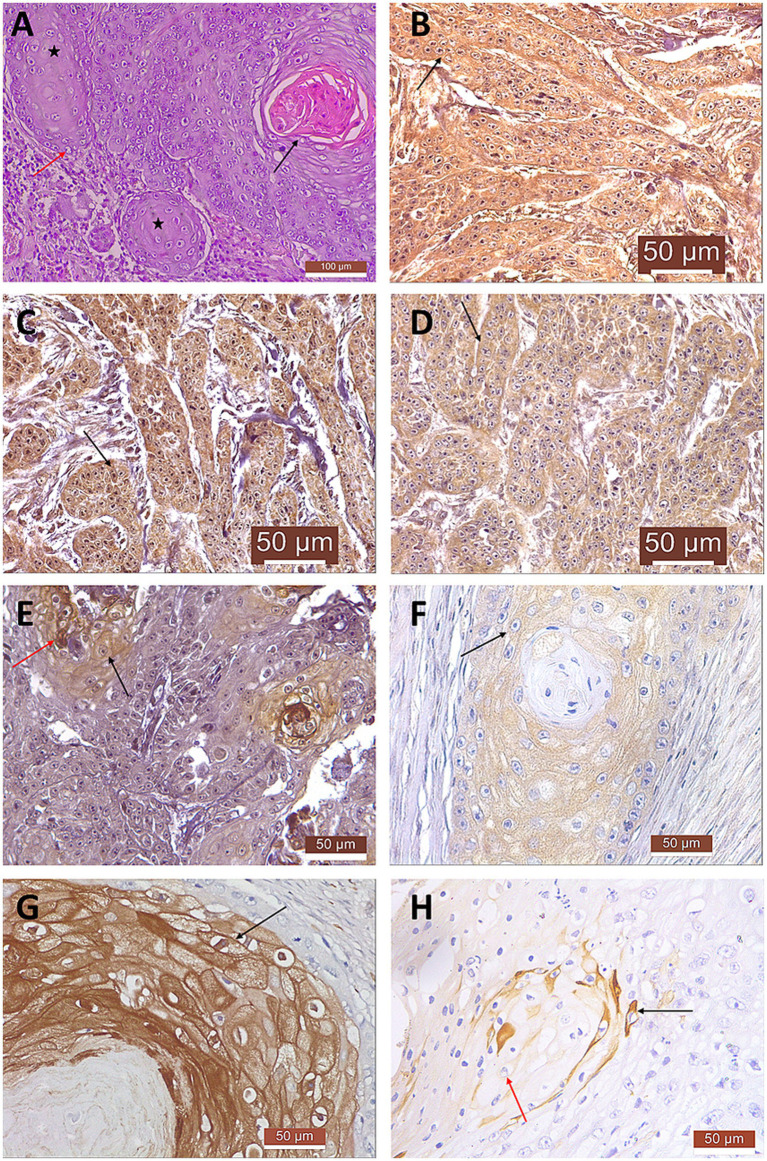
Histopathological characterization **(A)** and immunostaining of feline SCC **(B–H)**. **(A)** Moderately differentiated carcinoma exhibiting a diffuse, irregular cord-like infiltration within the deeper dermal layers (black stars), along with the presence of koilocytes (red arrow) and keratin pearl formation (black arrow); HE, 20X; **(B)** MMP-2 immunostaining showing moderate cytoplasmic and weak nuclear pattern (black arrow); 40X. **(C)** MMP-9 immunostaining appears as moderate cytoplasmic and nuclear pattern (black arrow); 40X. **(D)** MMP-13 immunostaining appears as moderate cytoplasmic and weak nuclear pattern (black arrow); 40X. **(E)** MMP-14 immunostaining is observed as moderate cytoplasmic and nuclear (black arrow) to intense cytoplasmic pattern (red arrow); 40X. **(F)** TIMP-2 immunostaining appears as weak to moderate granular cytoplasmic pattern (black arrow); 40X. **(G)** TIMP-3 immunostaining showing moderate to intense cytoplasmic and nuclear pattern (black arrow); 40X. **(H)** CK10 immunostaining appears as weak cytoplasmic (red arrow) to intense cytoplasmic pattern (black arrow); 40X.

The primary lesion observed was consisting in a moderately differentiated SCC, featuring a diffuse, irregular, cord-like infiltrate penetrating the deep dermis. The presence of numerous mitotic rates and multinucleated tumor cells is indicative of elevated proliferative activity. Keratin pearls confirm squamous differentiation, while koilocytes presence indicates a potential role of PV in tumorigenesis (Figure 1A). These architectural and cytologic traits collectively support a diagnosis of SCC.

### 3.3 PCR

Following the PCR assay, 6 out of 10 (60%) samples were found positive for PV infection. Among these, 5 were from female cats and one from a male. Regarding the breed, 2 samples were from Persian cats, while 4 from European cats. PCR positive samples were from both oral mucosa (2/3; 67%) and skin tissue (4/7; 57%) (Table 1).

### 3.4 Immunohistochemistry

To explore the reactivity of MMP-2/-9/-13/-14, TIMP-2/-3 and CK10 on tumor cells of feline SCC, the immunostaining was performed as follows: MMP-2 was performed on 10 samples, MMP-9 on 8 samples, MMP-13/-14, TIMP-2/-3 on 9 samples and CK10 on 5 samples.

The distribution and intensity of the immunostaining were consistent for the majority of analyzed samples, reflecting a predominantly cytoplasmic localization. Most samples exhibited moderate cytoplasmic immunoreactivity for MMP-2 (Figure 1B), MMP-9 (Figure 1C), MMP-13 (Figure 1D), and MMP-14 (Figure 1E), with a diffuse and granular pattern. In contrast, TIMP-2 (Figure 1F), and CK10 (Figure 1H) showed weaker cytoplasmic staining often limited to scattered cells, while TIMP-3 displayed an intense cytoplasmic immunoreactivity (Figure 1G). Nuclear staining was generally faint for MMP-2 and MMP-13, moderate for MMP-9, MMP-14, and TIMP-3, and absent for TIMP-2 and CK10.

*H*-score analysis using Mann Whitney *U* test indicated a higher expression of MMP-9, TIMP-3, and CK10 in cutaneous samples compared to oral ones, although these differences were not statistically significant (Supplementary Figures 1, 2). The only statistically significant difference was consisting in a higher TIMP-2 expression in PV-negative compared to positive samples.

Spearman’s rank correlation analysis (*ρ*) revealed significant biological relationships between protein expression evaluated by *H*-score (Supplementary Figure 3). Two significant positive correlation were observed between MMP-9 and TIMP-3 (*ρ* = 0.81, *p* = 0.015) and between MMP-2 and MMP-9 (*ρ* = 0.71, *p* = 0.046). The most notable inverse correlation was identified between MMP-2 and CK10 (*ρ* = −0.90, *p* = 0.037).

### 3.5 Western blot

Western Blot analysis was performed on 5 samples of SCC, 2 from skin and 3 from the oral cavity. Of these, 3 samples were positive for PV, while 2 samples were negative.

**Figure 2:**
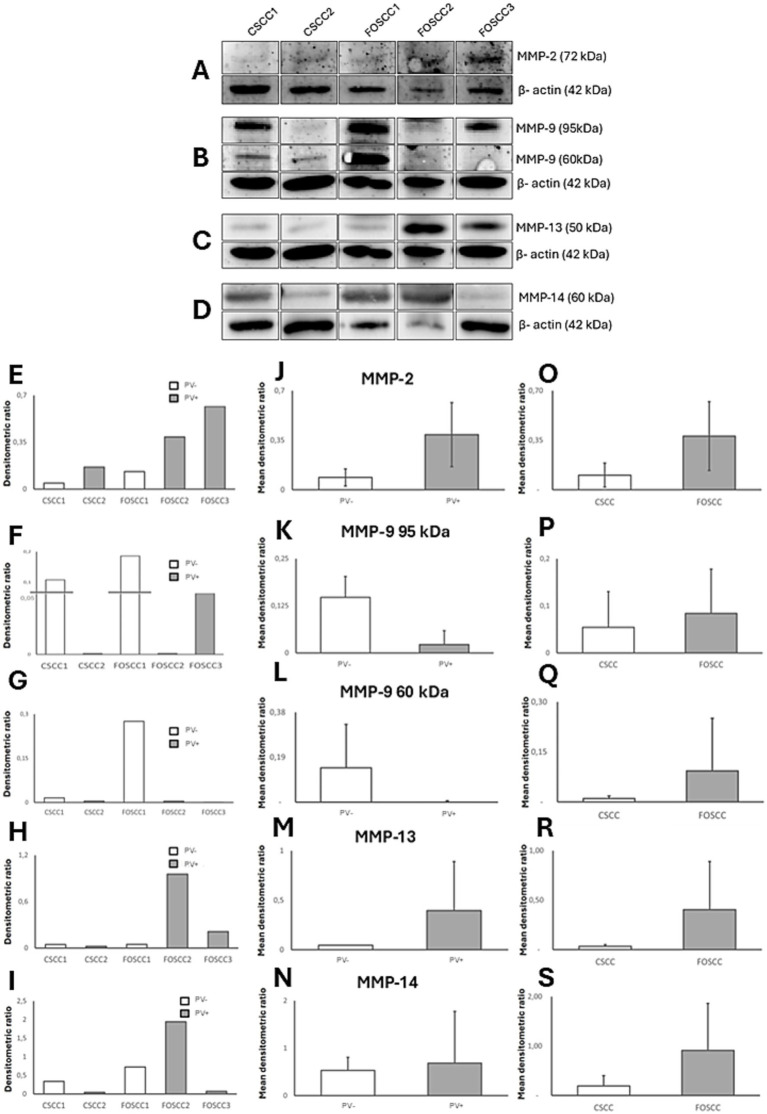
Western blotting analysis of MMP-2/-9/-13/-14 in papillomavirus positive (PV+) and papillomavirus negative (PV−) cutaneous squamous cell carcinoma (CSCC) and feline oral squamous cell carcinoma (FOSCC). **(A–S)** Western blotting and densitometric measurements of MMP-2/-9/-13/-14 in CSCC 1, 2 and FOSCC 1–3 samples. **(A–D)** Representative gels showing expression of MMP-2/-9/-13/-14. The blots were stripped and tested with anti-β-actin antibody to confirm comparable loading of proteins in each lane and allow normalization. **(E–I)** Individual densitometric values of MMP-2/-9/-13/-14 for each sample expressed as densitometric ratio with β-actin. **(J–N)** Mean densitometric values +/− standard deviations between PV+ and PV− samples. **(O–S)** Mean densitometric values +/− standard deviations between cutaneous and oral samples.

**Figure 3:**
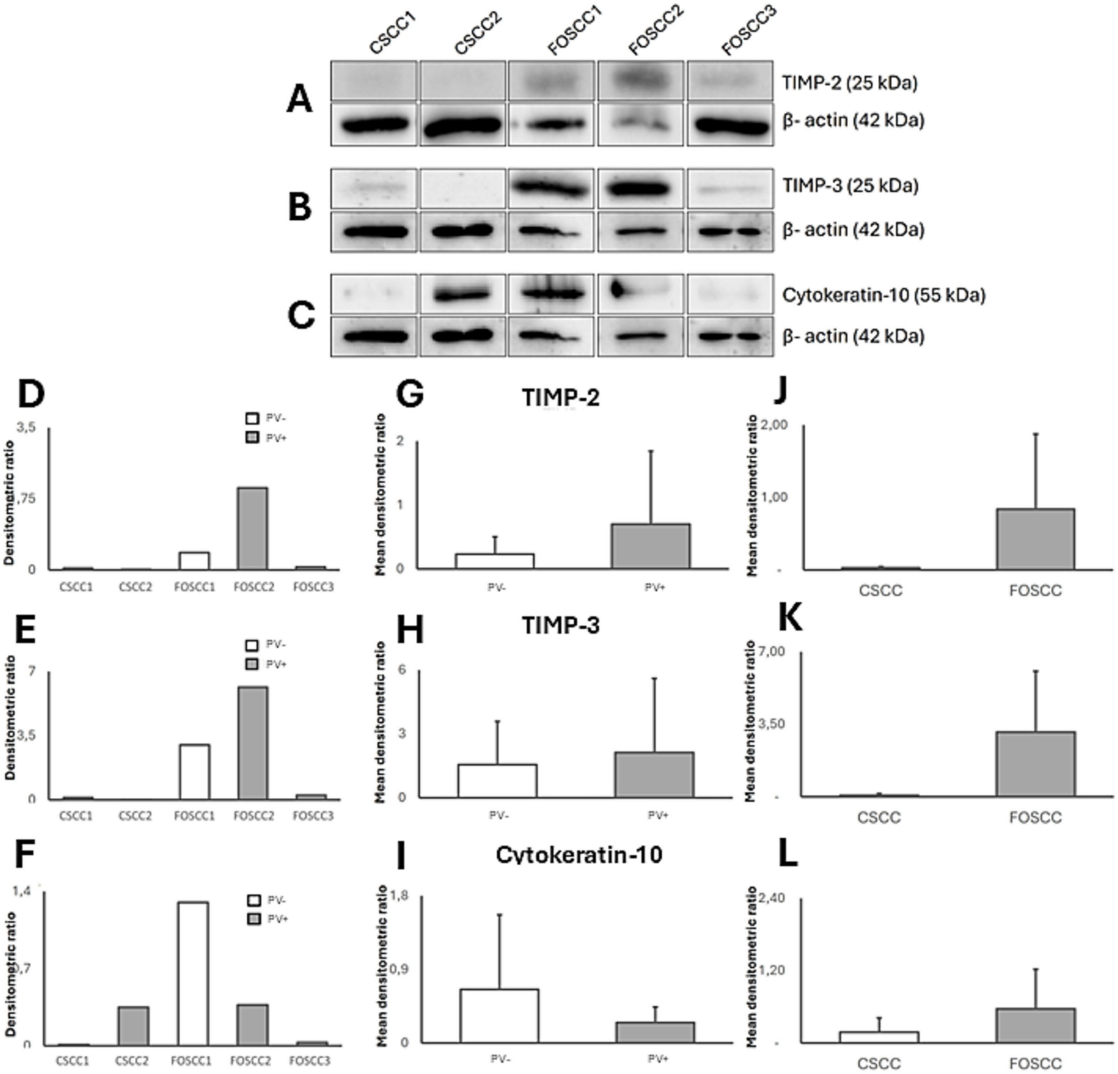
Western blotting analysis of TIMP-2/-3 and CK10 in papillomavirus positive (PV+) and papillomavirus negative (PV−) cutaneous squamous cell carcinoma (CSCC) and feline oral squamous cell carcinoma (FOSCC). **(A–L)** Western blotting and densitometric measurements of TIMP-2/−3 and CK10 in CSCC 1, 2 and FOSCC 1, 2, and 3 samples. **(A–C)** Representative gels showing expression of TIMP-2/-3 and CK10. The blots were stripped and tested with anti-β -actin antibody to confirm comparable loading of proteins in each lane and allow normalization. **(D–F)** Individual densitometric values of TIMP-2/-3 and CK10 for each sample expressed as densitometric ratio with β-actin. **(G–I)** Mean densitometric values +/− standard deviations between PV+ and PV− samples. **(J–L)** Mean densitometric values +/− standard deviations between cutaneous and oral samples.

Bands corresponding to expected molecular weights were detected for all tested antibodies—MMP-2 (~72 kDa), MMP-9 (~95/60 kDa pro/active forms), MMP-13 (~50 kDa), MMP-14 (~60 kDa) (Figure 2), TIMP-2 (~25 kDa), TIMP-3 (~25 kDa), and CK10 (~55 kDa) (Figure 3), confirming the specificity of IHC staining.

Densitometric analysis suggested possible trends, but was not pursued statistically due to the small sample size. When compared to the cutaneous samples, the oral samples showed higher expression for all investigated proteins. For the analyzed proteins, minor variations could be observed according to PV status, however this variation was not further analyzed due to limited samples (Figures 2, 3).

## 4 Discussion

This study investigated the expression of MMP-2/-9/-13/-14, TIMP-2/-3, and CK10 antibodies in feline SCC tissue samples.

OSCC is recognized as the most prevalent malignant tumor within the oral cavity of cats, accounting for approximately 60 to 75% of all oral tumors (23, 24). On the other hand, CSCC is one of the most frequently diagnosed skin tumors in felines, accounting for approximately 15–25% of all cutaneous neoplasms in cats (25).

CSCC is notably associated with environmental factors such as ultraviolet (UV) light exposure, particularly in non-pigmented areas of the skin (26). The presence of PV DNA in some cases of feline CSCC further emphasizes the role of viral infections as contributing factors. Research indicates that increased p16 protein expression correlates with UV protection in CSCC, suggesting a complex interaction between viral infections and UV exposure in tumor progression (27, 28). Conversely, feline OSCC displays different biological characteristics and is less likely to be associated with UV exposure. OSCC has been associated with viral infections such as feline PV (29). In our study, PV DNA was identified in 6 of 10 feline SCC cases, both skin and oral samples. This result reinforces the potential role of PV in the development of feline SCC. Future research should focus on identifying the specific feline PV types involved in these tumors.

To the authors knowledge, this is the first study to evaluate the expression of a panel of MMPs, TIMPs, and CK10 in feline SCC tissue samples using immunohistochemistry. Our findings revealed cytoplasmic MMP-2/-9/-13 and -14 staining in neoplastic cells, consistently with observations reported in human OSCC (30–32). This cytoplasmic presence is consistent with the classical biology of MMPs, reflecting their active synthesis in the form of pro-enzymes (zymogens) and storage in the vesicles of the Golgi apparatus and endoplasmic reticulum before being secreted into the extracellular environment for matrix degradation (33).

Our results are also in accordance with the findings reported by Daraban Bocaneti et al. (11, 12), describing cytoplasmic staining on tumor cells of bovine fibropapilloma using an anti-TIMP-2 and anti-TIMP-3 antibodies. This consistent cytoplasmic localization across different species and tumor types reinforces the role of TIMPs as key intrinsic regulators of the tumor microenvironment.

Furthermore, we identified nuclear reactivity for MMP-9, MMP-14, and TIMP-3, an observation that highlights the roles of these proteins in tumor progression. de Almeida et al. (34) emphasize that the nuclear translocation of MMPs is associated with functions independent of their extracellular proteolytic activity, such as transcriptional regulation, cell cycle control, and influencing genomic stability. Therefore, the nuclear localization of MMP-9 and MMP-14 may suggest a direct involvement in the genetic reprogramming of feline SCC tumor cells. Similarly, the presence of TIMP-3 in the nucleus has been documented to play a role in the regulation of apoptosis and cell survival, providing new insight into the mechanisms of intracellular control in SCC (35).

The strictly cytoplasmic localization of CK10 confirms its structural role as an intermediate filament, maintaining the integrity of the epithelial cytoskeleton without involvement in nuclear signaling (36).

The *H*-score is a quantitative measure to evaluate the expression levels of proteins or antigens in tissue samples. This scoring system integrates both the intensity of staining and the percentage of positively stained cells, providing a more comprehensive assessment than simple percentage scores alone (16). Following *H*-score analysis using Mann–Whitney *U* test, the only statistically significant result was noted on TIMP-2 expression, which was markedly higher in PV-negative samples compared to positive ones, consistent with da Silva Cardeal et al. (37), who demonstrated that co-expression of HPV-16 E6 and E7 oncoproteins negatively regulates TIMP-2 in human cervical cancer, suggesting possible similar mechanisms of virus-host interaction in the feline model.

Spearman’s correlation coefficient indicated a positive correlation between MMP-9 and TIMP-3, which may suggest that in feline SCC there is a compensatory response of the tumor microenvironment, attempting to balance the enzymatic activity of MMP-9 through the secretion of TIMP-3, an observation described in studies conducted on human SCC (38). Furthermore, the positive correlation observed between MMP-2 and MMP-9 seems to support the hypothesis of synergism between the two gelatinases in the process of tumor invasion, similar to the mechanisms described in human pathology (39). As for the inverse relationship between MMP-2 and CK10, it could reflect an association between invasive potential and loss of cellular differentiation, suggesting that reduced structural cohesion could facilitate the degradation of the extracellular matrix (ECM) (15). However, given the small size of the cohort, these correlations should be interpreted as biological trends that require validation on a larger sample study.

We acknowledge that the small and heterogeneous cohort, along with the varying number of samples available for certain markers (CK10), was primarily due to the limited quantity of available biological material within the archival blocks. These constraints are often inherent to investigations utilizing archival diagnostic specimens. The strong correlations observed, despite the limited sample size, provide a robust rationale for further validation in larger, multi-center cohorts.

Western blotting confirmed the specificity of the antibodies used, as bands at the expected molecular weights were detected for all markers tested. Densitometric analysis was performed, yielding results that indicated potential over-expression patterns of MMP-2, -9, -13, -14, and TIMP-2, -3 in feline OSCC versus CSCC. However, due to the limited sample size, these results were not subjected to statistical analysis. The observed patterns are consistent with prior research findings regarding the expression of aforementioned MMP and TIMP expression in oral compared to cutaneous SCC (40, 41). This validation, although performed on a pilot scale, reinforces the IHC observations and supports the translational relevance of the feline model, aligning with the complex molecular signatures documented in HHNSCC.

The limited sample size prevents generalization of these findings. Future studies involving larger sample populations approaches are needed to validate and reinforce these results, potentially elucidating the role of these markers in feline SCC pathogenesis. Specifically, integrated molecular approaches are necessary to validate these results and to clarify how specific feline PV species modulate tumor invasiveness and differentiation.

In conclusion, this pilot study is the first to characterize the expression of this specific subset of MMPs, TIMPs, and CK10 in feline SCC, integrating IHC and Western blot validation. These results establish a foundation for understanding the viral-molecular axis in feline oncology and reinforce the value of the feline model in comparative oncology research.

## Data Availability

The original contributions presented in the study are included in the article/Supplementary material, further inquiries can be directed to the corresponding author.
