# Supplementary material for: Immunohistochemical and western blot expression of MMPs, TIMPs, and cytokeratin 10 in feline squamous cell carcinoma
Source: Front Vet Sci. 2026 Apr 10;13:1787600. doi: 10.3389/fvets.2026.1787600 (PMC13105930; doi:10.3389/fvets.2026.1787600)
Supplement: Supplementary file 1 [file Data_Sheet_1.DOCX]

Supplementary Material

# Supplementary Figures and Tables

## Supplementary Figures
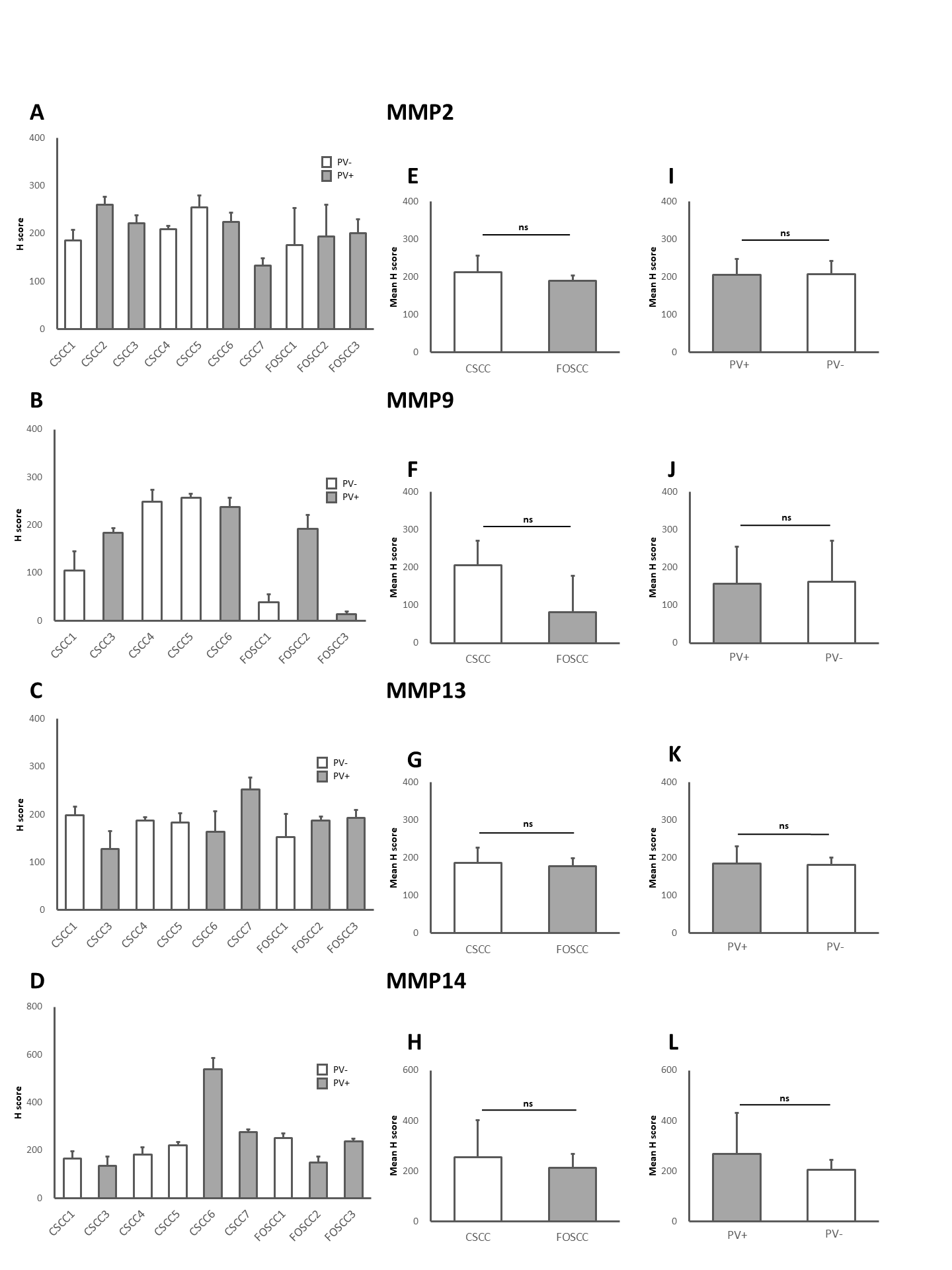


**Supplementary Figure 1.** Immunohistochemical expression of MMP-2/-9/-13/-14 in feline papillomavirus-positive (PV+) and papillomavirus-negative (PV-) cutaneous squamous cell carcinoma (CSCC) and oral squamous cell carcinoma (FOSCC) quantified by H score. (A-D) Individual mean H score values + standard deviation between different microscopical photos of each sample. PV+ and PV- represented by different bars pattern. (E-H) Mean H score values + standard deviation between CSCC and FOSCC samples showing an increased expression of MMP-2/-9/-13/-14 in cutaneous samples. (I-L) Mean H score values + standard deviation between PV+ and PV- samples showing an increased expression of MMP-14 in PV+ samples and similar expression of MMP-2/-9/-13 in all samples. Ns - p > 0,05; * - p < 0,05.


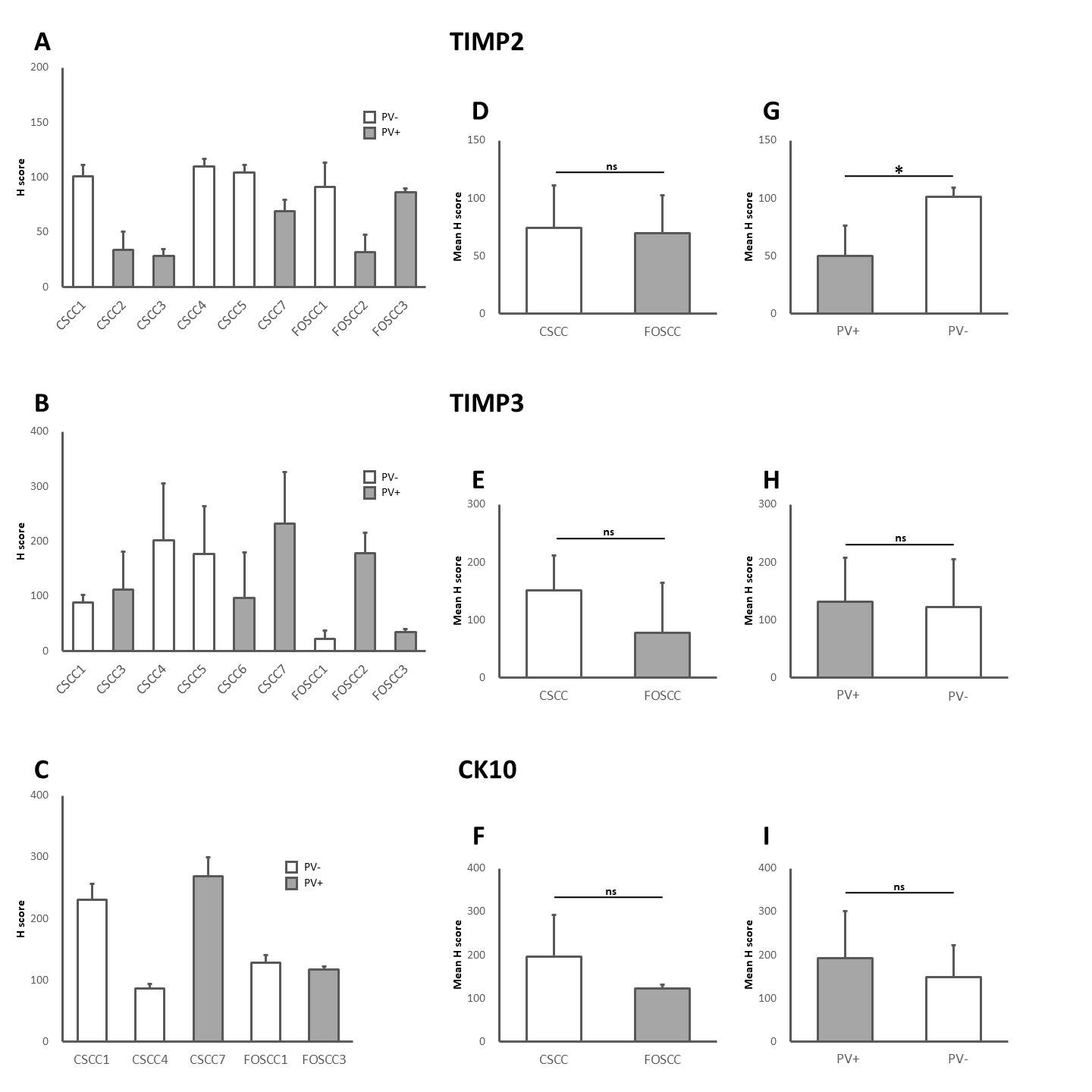


**Supplementary Figure 2.** Immunohistochemical expression of TIMP-2/-3, and CK10 in papillomavirus-positive (PV+) and papillomavirus-negative (PV-) cutaneous squamous cell carcinoma (CSCC) and oral squamous cell carcinoma (FOSCC) quantified by H score. (A-C) Individual mean H score values + standard deviation between different microscopical photos of each sample. PV+ and PV- represented by different bars pattern. (D-F) Mean H score values + standard deviation between CSCC and FOSCC samples showing an increased expression of TIMP-2/-3 and CK10 in cutaneous samples. (G-I) Mean H score values + standard deviation between PV+ and PV- samples showing an increased expression of TIMP-3 and CK10 in PV+ samples and an increased expression of TIMP-2 in PV- samples. Ns - p > 0,05; * - p < 0,05.


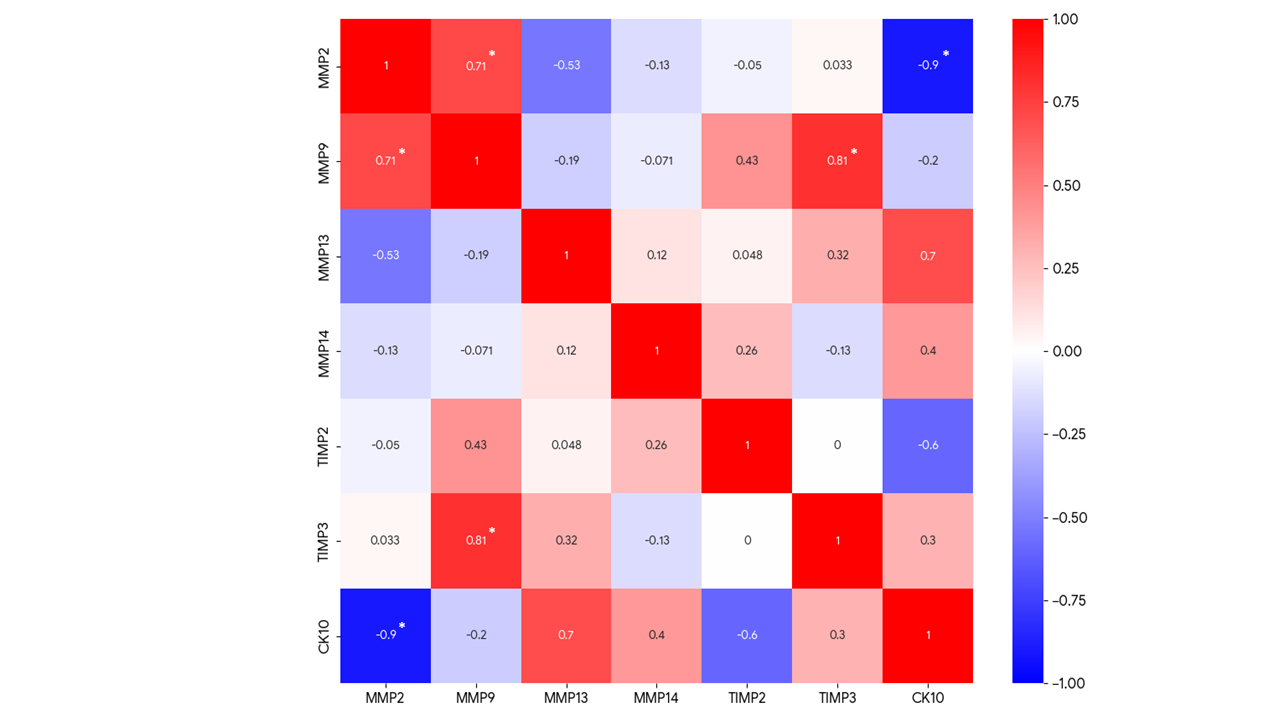


**Supplementary Figure 3.** Spearman’s correlation heatmap of H-score values for MMP-2/-9/-13/-14, TIMP-2/-3 and CK10 in feline SCC. The color scale ranges from blue (negative correlation, ρ = -1) to red (positive correlation, ρ = 1), with white representing no correlation (ρ = 0); * - p < 0,05.
